# The Relationship amongst Intervertebral Disc Vertical Diameter, Lateral Foramen Diameter and Nerve Root Impingement in Lumbar Vertebra

**DOI:** 10.5704/MOJ.1803.004

**Published:** 2018-03

**Authors:** MI Yusof, MN Hassan, MS Abdullah

**Affiliations:** Department of Orthopaedics, Universiti Sains Malaysia, Kubang Kerian, Malaysia; ^*^Department of Orthopaedics, Hospital Raja Perempuan Zainab II, Kota Bharu, Malaysia; ^**^Department of Radiology, Universiti Sains Malaysia, Kubang Kerian, Malaysia

**Keywords:** intervertebral disc, lateral foramen, root compression, lateral stenosis

## Abstract

**Introduction:** The vertical diameter of the foramen is dependent upon the vertical diameter of the corresponding intervertebral disc. A decrease in disc vertical diameter has direct anatomic consequences to the foraminal diameter and area available for the nerve root passing through it. This study is to establish the relationship amongst the intervertebral disc vertical diameter, lateral foramen diameters and nerve root compression in the lumbar vertebra.

**Materials and Methods:** Measurements of the study parameters were performed using sagittal MRI images. The parameters studied were: intervertebral disc vertical diameter (DVD), foraminal vertical diameter (FVD), foraminal transverse diameter (FTD) and nerve root diameter (NRD) of both sides. The relationship between the measured parameters were then analyzed.

**Results:** A total of 62 MRI images were available for this study. Statistical analysis showed moderate to strong correlation between DVD and FVD at all the lumbar levels except at left L23 and L5S1 and right L3L4 and L4L5. Correlation between DVD and FTD were not significant at all lumbar levels. Regression analysis showed that a decrease of 1mm of DVD was associated with 1.3, 1.7, 3.3, 3.3 and 1.3mm reduction of FVD at L1L2, L2L3, L3L4, L4L5 and L5S1 respectively.

**Conclusion:** Reduction of DVD was associated with reduction of FVD. However, FVD was relatively wide for the nerve root even with complete loss of DVD. FTD was much narrower than the FVD making it more likely to cause nerve root compression at the exit foramina. These anatomical details should be given consideration in treating patients with lateral canal stenosis.

## Introduction

There are 23 intervertebral discs in the entire spine contributing to approximately 25% of the vertical diameter of the spine^1^. With increasing age, disc water content decreases especially in the nucleus pulposus leading to reduction of nucleus hydrostatic pressure and reduction of intervertebral vertical diameter^2^. The intervertebral foramen or the lateral canal is located posterior to the disc. It is the exit route for the spinal nerve root from the spinal canal after it leaves the spinal cord. The intervertebral foramen also transmits the spinal nerves, spinal arteries and veins, the recurrent meningeal nerves and lymphatics. This foramen is unique in comparison to other foramina of the body due to its boundaries consisting of two movable joints: the ventral intervertebral joint and the dorsal zygapophyseal joint^3^. The proximity of these joints increases the susceptibility for lateral foramen narrowing from structural alterations. The lateral foramen is oval in shape, the roof formed by the inferior aspect of the pedicle of the superior vertebra, the ligamentum flavum, pars interarticularis and the zygapophyseal joint. The floor of the foramen is the superior part of the pedicle of the inferior vertebra, the intervertebral disc and the postero-superior margin of the inferior vertebral body^4^.

The vertical diameter of the foramen is dependent upon the vertical diameter of the corresponding intervertebral disc. With aging there is a natural tendency toward disc degeneration and loss of disc vertical diameter. This decrease in disc vertical diameter has direct anatomic consequences on the foraminal diameter and area available for neurovascular structures passing through it. Decrease of foraminal diameter may cause compression to the nerve root that crosses the foramen^5^. Sohn *et al* showed that there was a significant correlation between disc vertical diameter and foraminal transverse diameter in the cervical spine^6^. Knowledge about the correlation between changes in disc vertical diameter and foraminal diameter with possibility of nerve root compression in the lumbar spine might give a new insight into the treatment required for the patients.

The objectives of this study were to establish the relationship amongst the intervertebral disc vertical diameter, lateral foramen diameter and the nerve root impingement in the lumbar vertebra using MRI.

## Materials and Methods

This is a cross-sectional study that involved analysis data from the magnetic resonance imaging (MRI) of the lumbar sacral spine from the electronic library (picture, archiving and collection, PAC system) in the Department of Radiology. The subjects for this study were randomly chosen (systematic random sampling) from those who had undergone MRI for low back pain between 2004 and 2008. Patients with congenital spinal deformity, lumbosacral transitional vertebrae, primary tumour, metastases, traumatic injury and infection were excluded from the study. Anterior-posterior (AP) and lateral lumbar radiographs were taken in suspicious cases (congenital abnormalities, transitional vertebra, etc.). All abnormal cases were excluded from our study.

**Fig. 1:**
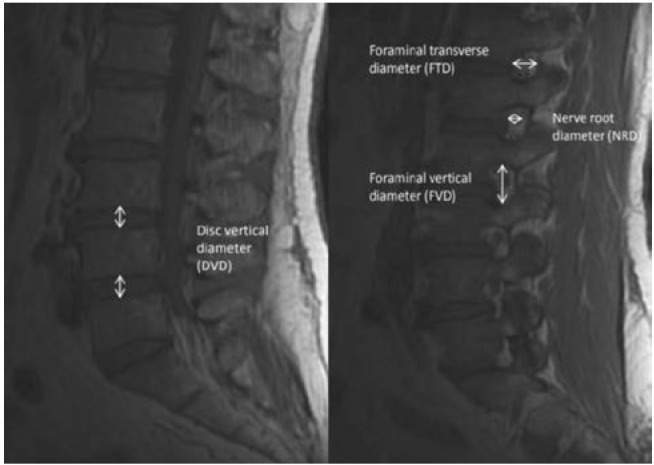
Methods of measuring disc vertical diameter (DVD), foraminal transverse diameter (FTD), foraminal vertical diameter (FVD) and nerve root diameter (NRD).

Measurements of the study parameters were performed using sagittal MRI (T1) images based on axial view. The parameters studied were: (i) intervertebral disc vertical diameter (DVD), measured at the centre of the intervertebral disc, (ii) foraminal vertical diameter (FVD) of both sides, measured at the keyhole view of the lateral foramen by the distance between the pedicles, (iii) lateral foraminal transverse diameter of both sides (FTD), measured at the superior margin of intervertebral disc to lower margin of posterior facet, (iv) nerve root diameter (NRD) of both sides, measured in the keyhole of the lateral foramina (Fig. 1). Excess area for the nerve root (ESN) was calculated by subtracting the foraminal diameter and nerve root diameter. Measurements were made at every disc level from L1L2 to L5S1. Analysis of correlation of the studied parameters was performed using Bivariate correlation method and Regression analysis was used to correlate between reduction in the disc vertical diameter (DVD) and diameter changes occurring in the lateral foramen.

This study was approved by our institution’s research and ethics committee.

## Results

A total of 62 MRI images were available for these studies with 620 lateral foramina measured with seven different parameters were measured at each foramen. The mean age of sample was 43 year old, ranged from 16 years to 70 years. They were 30 (48.4%) males and 32 (51.6%) females involved.

At L1L2 lumbar vertebra, the mean DVD, right RD, right FVD, right FTD, left RD, left FVD and left FTD were 6.6 (1.4), 3.4 (0.5), 18.2 (2.3), 8.3 (1.3), 3.4 (0.5), 18.4 (1.0) and 8.0 (1.3) mm respectively. All the measurements obtained from this study were summarized in Table I. There were no significant differences between the measurements of the right and left RD, FVD and FTD at all lumbar levels.

**Table I:** Mean disc vertical diameter, nerve root diameter, foraminal vertical diameter and transverse diameter of the 62 studied patients based on MRI measurement

Lateral foramen							
Level	Disc vertical diameter, DVD (mm)	Nerve root diameter NRD (mm)		Vertical diameter, FVD (mm)		Transverse diameter (mm)	
		Rt	Lt	Rt	Lt	Rt	Lt
L1L2	6.6 (1.4)	3.4 (0.5)	3.4 (0.5)	18.2 (2.3)	18.4 (1.9)	8.3 (1.3)	8.0 (1.3)
L2L3	7.8 (1.4)	3.6 (0.6)	3.5 (0.5)	19.6 (2.2)	19.6 (2.1)	7.9 (1.5)	7.5 (1.1)
L3L4	8.7 (1.6)	3.6 (0.6)	3.8 (0.7)	18.9 (2.3)	19.0 (1.9)	7.8 (1.5)	7.8 (1.6)
L4L5	9.0 (1.8)	3.6 (0.7)	3.6 (0.6)	17.8 (2.3)	17.7 (2.5)	7.4 (1.4)	7.5 (1.5)
L5S1	8.7 (2.0)	3.6 (0.7)	3.8 (0.9)	16.4 (2.7)	15.9 (3.1)	7.4 (1.8)	7.6 (1.7)

Statistical analysis showed positive correlation between DVD and FVD at all the lumbar levels with moderate to strong correlation except at the left L23 and L5S1 levels and right L34 and L45. Correlation between DVD and FTD were not significant at all levels of lumbar spine (Table II). Regression analysis showed strong correlation (t=5.94, p<0.001 at L5S1, t=2.13, p<0.05 at L4L5) between changes in the DVD and FVD at L1L2, L2L3, L4L5 and L5S1. FVD was dependent on the changes of DVD and this correlation is represented by the equation;

**Table II:** Correlation between Intervertebral Disc (DVD) and Lateral foramen vertical diameter (FVD) and Lateral foramen transverse diameter (FTD)

Level	Correlation between Disc Vertical diameter (DVD) and Lateral Foraminal Vertical diameter (FVD) and transverse diameter (FTD)			
	FVD		FTD	
	Rt	Lt	Rt	Lt
L1L2	0.500	0.439	.047	0.042
L2L3	0.396	0.280	-0.028	-0.162
L3L4	0.230	0.412	0.063	0.076
L4L5	0.265	0.464	0.158	0.046
L5S1	0.608	0.02	-0.099	0.241

0 < |r| < .3 weak correlation

.3 < |r| < .7 moderate correlation

|r| > 0.7 strong correlation

y=ax +b

y= FVD

x= DVD

a and b = constants

Using linear regression analysis, predicted measurements of foraminal vertical diameter with 25%, 50%, 75% and 100% (complete) loss of disc vertical diameter reduction were described in Table III.

**Table III:** Predicted area available for the nerve root at the lateral foramen of the lumbar spine with percentages of disc vertical diameter loss (without listhesis)

Level	Predicted foraminal vertical diameter, FVD (mm)				
Disc height loss (%)	0	25%	50%	75%	100%
L1L2	18.2	16.6	15.2	13.9	12.6
L2L3	19.6	18.2	17.0	15.9	14.7
L3L4	18.9	17.9	17.2	16.6	15.9
L4L5	17.8	16.7	16.1	15.4	14.7
L5S1	16.4	14.6	12.8	11.1	9.4

## Discussion

In this study, the mean intervertebral disc vertical diameter (DVD) at L1L2 was 6.6 (1.4) mm. The mean disc vertical diameter measurements showed an increasing trend from L1L2 to L4L5 with measurement of 9.0 (1.8) mm. The highest disc diameter was found to be at L4L5 level. The measurement at L5S1, however, showed reduction of disc vertical diameter to 8.7 (2.0) mm. The pattern of disc vertical diameter measurement was fairly similar compared to other studies which showed reduction of L5S1 disc vertical diameter. However, their disc vertical diameter was reported to be higher as the measurements were performed in Caucasians^7^. The Caucasian population probably had higher measurements in other parameters as well as shown in other studies^8,9^.

**Fig. 2:**
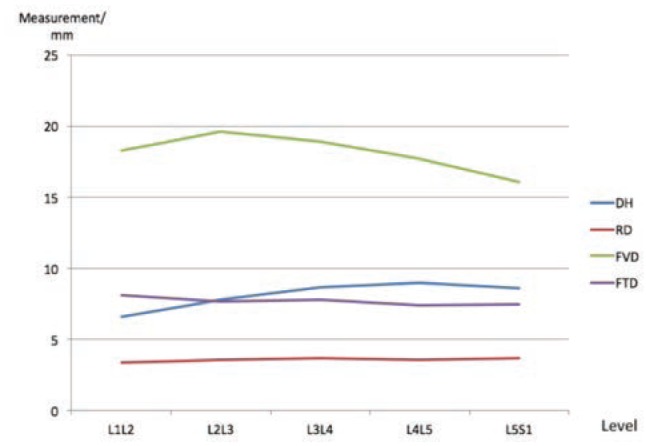
Measurements of the disc vertical diameter (DVD), nerve root diameter (RD), foraminal vertical diameter (FVD) and foraminal transverse diameter (FTD) of the lumbar spine.

The foraminal vertical diameter generally showed downward trends from proximal to distal lumbar spine. Both the mean vertical diameters (FVD) and transverse diameters were found to be smallest at L4L5 (17.8, 7.5mm respectively) and L5S1 (16.1, 7.5mm respectively) levels compared to the above levels. The mean FVD was the highest at L2L3 (19.6mm) and the mean FTD was the widest (8.2mm) at L1L2 level (Fig. 2). The mean nerve root diameter (RD) at the lower lumbar was also noted to be bigger than that at the upper levels. The discrepancy between nerve root diameter and lateral foraminal diameters would indicate relatively increased risk of nerve root compression as it exits from the lateral canal. These findings give additional explanation to the higher incidence of spinal stenosis at the lower lumbar region compared to upper lumbar levels.

Analyses of the measurements data showed positive correlation between DVD and FVD in all lumbar spinal segments. However, no positive correlation was observed between the DVD and FTD in this study (Table II).

Sohn *et al* did similar morphometric study on cadaveric cervical spine and found that there was no positive correlation between DVD and FVD^6^. However, positive correlation between the disc vertical diameter and lateral foramen FTD was observed. This indicates the anatomical difference between cervical and lumbar spines. The cervical spine pedicles are more oblique and longer compared to lumbar spines. Translation of cervical vertebra over the caudad vertebra probably occurs with reduction of disc vertical diameter. This was not observed in the lumbar spines in this study.

**Fig. 3:**
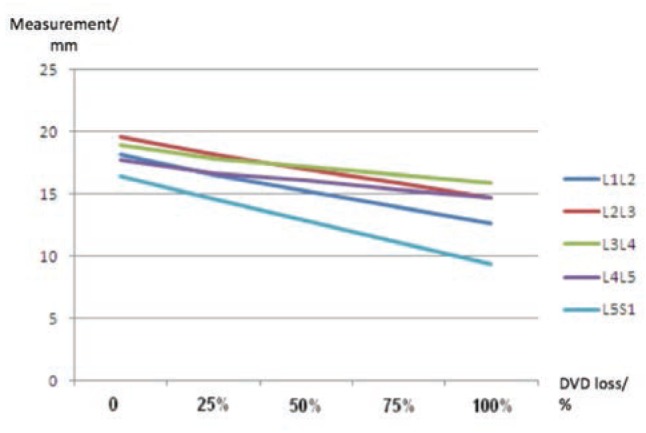
Predicted measurements of foraminal vertical height with vertical disc diameter loss at every lumbar spinal level.

Reduction of disc vertical diameter was followed by the foraminal vertical diameter (Fig. 3). The FTD, however, was not affected by disc vertical diameter reduction. With disc vertical diameter reduction of 25% and 50%, the foraminal vertical diameters at L1L2, L2L3, L3L4, L4L5 and L5S1 were between 14.6 to 18.2mm and 12.8 to 17.2mm respectively (Table III). Even with total loss of disc vertical diameter, the foraminal vertical diameter was still more than the nerve roots size. The FVD measurements were between 9.4 and 15.9 mm at the lumbar spines, more than two times larger than the FTD. Comparing to the nerve root diameter, there was still excess of area available for the nerve root to pass through the lateral foramina. This indicated that nerve root impingement in symptomatic patients were not contributed significantly by the reduction in the vertical diameter of the lateral foramen.

Clinically, these findings suggest that impingement of the nerve root by prolapsed disc is more likely secondary to reduction of foraminal transverse diameter (FTD) of the lateral foramen, rather than the vertical diameter (FVD). Therefore, discectomy or laminotomy alone is probably adequate for nerve root decompression in young patients who have minimal degenerative changes as these would improve the FTD. Some patients may have symptoms of nerve compression due to chemical radiculopathy without radiological evidence of compression. These patients therefore have normal transverse diameter and would not benefit from decompressive surgical procedure.

The belief that placement of implant or bone graft in interbody fusion procedure (posterior lumbar interbody fusion, PLIF) and implantation of interspinous devices would primarily decompress the nerve root by increasing FVD probably needs reconsideration. Our findings showed that reduction of disc height does not cause nerve root compression as the area for the root was in excess. This procedure, however, definitely improves vertical diameter of the lateral foramen and reduces facet joints loading. In this situation, decompression of the nerve root would be achieved with improvement of vertical diameter in the presence of vertical stenosis. On the other hand, foraminal transverse diameter (FVD) would not be improved with this procedure as there was no correlation between DVD and FTD shown in this study. Therefore, nerve root impingement due to reduction of FTD must be dealt with directly by restoring the normal transverse diameter, for instance by removing the osteophytes or undercutting hypertrophic facets. Transforaminal lumbar interbody fusion (TLIF) procedure is particularly advantageous for transverse decompression.

## Conclusion

There was positive correlation between the intervertebral disc vertical diameter and the foraminal vertical diameter in lumbar vertebra. Reduction of disc vertical diameter would be associated with reduction of foraminal vertical diameter. Foraminal transverse diameter is much narrower than the nerve root diameter making it more likely to cause nerve root compression at the exit foramina. These anatomical details should be given consideration when treating patients with lateral canal stenosis.

## Conflict of Interest

The authors declare no conflict of interest.
